# The impact of digital healthcare and teledentistry on dentistry in the 21st Century: a survey of Hungarian dentists

**DOI:** 10.1186/s12903-023-03770-w

**Published:** 2023-12-19

**Authors:** Orsolya Németh, Eszter Uhrin, Edmond Girasek, Julianna Boros, Zsuzsa Győrffy

**Affiliations:** 1 Department of Community Dentistry, Faculty of Dentistry, Semmelweis University, Budapest, Hungary; 2 Institute of Behavioral Sciences, Faculty of Medicine, Semmelweis University, Budapest, Hungary

**Keywords:** Teledentistry, Digital dentistry, Digital workflow

## Abstract

**Background:**

The era of digitalization has arrived in the field of dentistry. Teledentistry (TD), the use of digital solutions in dentistry, is already used in practice; however, only some possibilities are considered. During the COVID-19 pandemic, remote patient monitoring and patient communication had to be solved with TD, thus causing a rapid spread of new tools. In addition to digital workflows, patient communication, AI, and online forums are also available.

**Methods:**

An online self-administered survey was developed for the study. The Hungarian Medical Chamber contacted potential respondents in a newsletter or e-mail. The Evasys survey system was used. The weighting procedure was executed for gender, age group, and type of settlement. A digital dental index variable was created and built with a linear regression model as a dependent variable. Explanatory variables are advantages, disadvantages, what would be necessary, experienced needs from the patients, and age.

**Results:**

A total of 171 dentists completed the survey. The best-known digital technologies are online conferences (96.5%), E-prescriptions (94.6%), and digital impressions (86.0%). Unawareness is the highest in the field of artificial intelligence in dentistry (50.5%), store-forward solutions (43.5%), and real-time solutions (41.8%). The digital dental index is 14.24 (standard deviation (SD) = 5.5), with a high power of the model.

**Conclusion:**

Hungarian dentists need to be made aware of all the possibilities of TD. In addition to digital workflows, store-forward and real-time solutions can be beneficial to substitute face-to-face visits. TD can be used effectively to monitor oral cavity changes and develop dental confidence and proper oral care habits. Our survey suggests that it is necessary and inevitable to integrate TD into both graduate and postgraduate education, which may form the basis of primary health care in the next decade.

## Background

Vibrant digital healthcare - from e-patients to digital workflow - redefines healthcare in the 21st century. Telemedicine services can replace face-to-face visits; therefore, the most significant emphasis is placed on doctor-patient communication, such as store-forward, remote patient monitoring, patient follow-up, discussion, and evaluation of findings [1]. Teledentistry (TD), being part of telemedicine, deals with modern information technology in dentistry. It has yet to be implemented entirely in everyday dental practice [2]. However, with the development of TD, educational materials to support the training of colleagues were limited [3, 4].

The conventional dental workflow is disappearing and being replaced by digital dental workflow. Imaging devices, including intraoral scanners, extraoral scanners, and cone beam computed tomography (CBCT), can transform the shape of a patient’s tissues into three-dimensional (3D) data. With the help of computer-associated milling technologies and 3D printing, it is possible to create surgical implant guides according to digital planning or dental prostheses, even within one hour. Digitalization in the dental industry is a revolution that will continue to shape the future of dentistry. However, let us remember the fields of dentistry where digitization accelerates patient care through digital patient monitoring with applications about prevention and communication as a novel approach [5].

During the pandemic, several dental clinics used services based on information communication technology to meet the changing needs of e-patients. This led to store-forward and real-time solutions focusing on doctor-patient communication [6]. The spread of COVID-19 forced dental facilities to reduce their services to emergency patient care. TD helped them with the e-triage system, early (tele)diagnosis of chronic oral diseases, and dental prevention [7].

Consequently, traditional communication between dentists and patients has been replaced by the era of e-patients. Patient data and medical reports have been integrated into telemedicine services to facilitate accessibility of the information [8, 9].

Indeed, the usage of these services is not only influenced by legal but also financial questions. Protecting patient medical data and cybersecurity has become an important and unsolved issue. Telemedicine services became a determinative part of working hours, which led to extra time-consuming and non-honored Sect. [10].

The landscape of TD research in Hungary still needs to be explored, particularly in the context of its implementation and comprehension among dental professionals. Notably, our study is an original contribution to the existing body of knowledge by delving into the nuances of TD utilization among Hungarian dentists. The novelty of our research lies in its focus on assessing the awareness, utilization, and perceptions of diverse TD tools, shedding light on a previously uncharted territory within the Hungarian dental sphere.

Our research aims to assess the knowledge and affinity of Hungarian dentists for TD. In addition, identifying and mapping the existing knowledge gaps can aid us in tailoring our teaching efforts to emphasize how TD can benefit dentistry.

## Materials and methods

### Study design and participants

An online survey was conducted as part of “E-doctors and E-patients in Hungary” research among Hungarian dentists (Number of ethical approval: IV-10927-1 TUKEB). The online survey was a self-administered form available for three months. The research team developed the questionnaire, and an important aspect was that the obtained results could be compared with our population-based survey.

### Survey

The dentistry-associated part of the survey has five main sections (sociodemographic, dental work, digital technologies, and digital healthcare-related concerns) and 84 questions.

The pretesting procedure was conducted by eight dentists in the Department of Community Dentistry, Semmelweis University, Budapest. They were from different dental fields and varied in age (23–40).

### Data collection

The respondents were contacted with the help of the Hungarian Medical Chamber in a newsletter or a personal e-mail invitation letter. The population was Hungarian dentists who provided a valid e-mail address to the chamber. The participants could access the questionnaire through a link. Nonresponse was handled by resending the survey. After that, we closed the survey and checked for duplicated fillings but found none. We used the Evasys survey system, which does not remember IP addresses; thus, respondents’ identification is impossible, so it complies with the GDPR requirements.

The four-dimension corrective weighting procedure was executed by gender, age, city (Budapest or not), and specialization (specialized or not). After weighing these four dimensions, the data in these variables estimates the Hungarian dentists.

### Statistical analysis

SPSS [11] software was used for the statistical analysis. To avoid missing data, every outcome is listed with the number of received answers (valid percent). We used descriptive statistics to present demographic data (frequencies, medians (M), means, crosstabs). The P-value below 0,05 was considered significant.

We created a digital dental index variable, and a linear regression model was built on the digital dental index variable as a dependent variable, where the explanatory variables are advantages, disadvantages, what would be necessary, experienced needs from the patients, and age. The index ranges between 0 and 27.

A digital dental index (DDI) was calculated as a summary of the following variables:

- How many have you recommended of these: applications, social media groups, social media sources;
- How many do you know: e-Health Care Cloud Hosting, teledentistry-store-forward, teledentistry-real-time applications;
- How many do you use (value of at least 3): e-Health Care Cloud Hosting, teledentistry-store-forward, teledentistry-real-time applications;
- How many do you want to use (value of at least 3): e-Health Care Cloud Hosting, teledentistry-store-forward, teledentistry-real-time applications;
- How many do you know: intraoral scan, 3D design, 3D printing, 3D surgical template;
- How many do you use (value of at least 3): intraoral scan, 3D design, 3D printing, 3D surgical template.

A linear regression model was built for the mapping of its influencing factors using the following variables:

- How many advantages do you see in digital healthcare solutions?
- How many disadvantages do you see in digital healthcare solutions?
- How many things would you need to use digital healthcare solutions?
- How many solutions have you experienced need from your patients?
- Respondent age.

## Results

A total of 6,894 dentists were contacted, from which 171 (0,025%) dentists completed the survey. All demographic data about the participants are summarized in Table 1. Out of 171 dentists, 40 men and 131 women completed the study, with an age distribution of 73 below 40 years old (42.7%) and 98 older than 40 years (57.3%). Most of the participants (99.4%) were working primarily in Hungary. 35.5% work in the capital city (Budapest), and 64.5% work in the countryside. The majority had one or more specialties (84.6%), working as a dental specialist. Regarding the main workplace, 33.1% worked in a private praxis, and 14.9% worked at a university. Regarding hours spent in patient care, most participants (50.7%) worked 30 or fewer hours weekly. 13.8% treated patients between 50 and 149 km, and 2.9% treated patients with a distance > 150 km. The use of the Internet among Hungarian dentists is summarized in Table 2.

**Table 1:** Demographic data of participants according to age, location, specialties, and their praxis

	*Number of participants*	*Frequency*
***Age***		
*25–29*	13	7.7
*30–34*	24	13.9
35–39	21	12.4
40–44	18	10.2
45–49	21	12.2
50–54	18	10.4
55–59	17	10.1
60–64	13	7.4
65–69	10	6.0
70+	17	9.6
***Location***		
*Budapest (capital)*	82	48.0%
*Pest County*	15	8.8%
*Countryside*	74	43.3%
***Number of specialties***		
*0*	26	15.4%
*1*	80	47.1%
*1+*	66	37.5%
***Hours spent in patient care***		
*≤ 30*	85	50.7%
*31–40*	62	37.0%
*> 40*	21	12.3%
***Distance from the dentist (km)***		
*0–49*	143	83.2%
*50–149*	24	13.8%
*> 150*	5	2.9%

**Table 2:** Use of the Internet among dentists

***How often do you use the Internet for your everyday work?***		
*Multiple times a day*	126	73.6%
*Daily*	33	19.5%
*Multiple times a week*	8	4.8%
*Never*	4	2.1%
***What do you use the Internet for? (Multiple choice)***		
*Research, training*	138	80.4%
*Patient communication*	118	68.8%
*Social media*	118	68.3%
*Planning*	88	51.3%
*Education*	66	38.4%
*Professional entertainment*	62	35.8%

The best-known digital technologies are online conferences (96.5%), E-prescriptions (94.6%), and digital impressions (86.0%). Unawareness is the highest in the field of artificial intelligence in dentistry (50.5%), store-forward solutions (43.5%), and real-time solutions (41.8%) (Table 3).

**Table 3:** The known and used technologies in the dental field

Question	I never use		I do not use		I use sometimes		I use		I use it every day		All participants
***Do you suggest websites to your patients?***	42	24.4%	41	24.1%	52	30.6%	26	15.2%	10	5.7%	171
***Do you suggest mobile applications to your patients?***	89	51.9%	45	26.1%	29	17.0%	5	2.7%	4	2.2%	171
***Do you suggest social media platforms to your patients?***	106	51.4%	32	18.4%	23	13.6%	8	4.6%	4	2.1%	172
***What do you think of the medical use of the Internet among patients?***	1	0.8%	12	6.7%	87	50.7%	51	29.8%	21	12.0%	172
***How would you rate your knowledge of internet use compared to your patients?***	5	3.2%	4	2.4%	48	27.6%	67	38.9%	48	27.9%	172

Investigating the intention to use digital communication systems (DCS), such as e-Health Care Cloud Hosting, store-forward and real-time solutions, and smartphone applications, it was found that 91.7% would like to apply these tools to their everyday dental practice in the future; however, on average, they know median (M) = 3.0 but use only half of it (M = 1.0). One out of 8 dentists says they use none of the digital communication solutions listed above (12.8%, n = 22).

Younger dentists know more DCS than older generations, but in terms of use, we do not see a clear correlation with age. According to gender, there is no statistically significant difference either in how many communication tools they know or how many they use. Regarding future needs, women and men would like to use M = 2 digital tools. In the case of the workplace, dentists working at public dental offices are less likely to use (M = 1, n = 18), whereas those working at clinics and universities are the most likely to use (M = 3, n = 15 and M = 2, n = 26, respectively). Dentists from the capital and villages use M = 2 DCS (n = 61; n = 13), respectively.

We asked the dentists about dental digital sources (DDS): webpages, social media, and smartphone applications. More than ¾ of them have already recommended DDS to their patients. 78.8% (n = 136) suggested at least one type of DDS, and 31.6% (n = 54) recommended all three. 35.6% (n = 46) of the respondents did not use any dental applications. Recommendations are not significantly associated with age, sex, or place of work; however, we see differences in the type of settlement. In the capital, other cities, and villages, they recommended M = 2 platforms out of three, whereas they recommended M = 1 in the county capital cities’ dentists.

Digital workflow (3D planning, 3D printing, 3D surgical guide) is commonly known by dentists (89.1%; n = 153), but 71% (n = 122) have yet to use it. In the future, 77.8% (n = 134) would like to try at least one of these tools. 36.9% (n = 64) wanted to use all digital workflow tools. 10.9% of the respondents (n = 19) needed to be aware of digital workflows. There is a significant correlation between age and the answers: the younger generation tends to know and use more and would also like to use it in the future. There are two breakpoints in age regarding the knowledge of digital workflow tools: at the ages of 55 and 70, there is a significant drop in knowledge compared to young dentists. Women use fewer digital workflow tools than men (M = 0; mean = 0.5, n = 105; M = 0; mean = 1, n = 68, respectively). There is no significant difference in sex regarding the number of known technologies or their use in the future. Regarding the type of workplace, dentists from clinics (M = 0; mean = 1.0, n = 15) and universities (M = 1; mean = 1.2, n = 26) use more digital workflow tools than dentists from public dental offices (M = 0; mean = 0.2, n = 18). In the capital, dentists do not use digital workflow tools; however, in this case, they tend to use more than one (M = 0; mean1.1; n = 61). In villages, only a few dental offices use digital workflow tools (M = 0; mean = 0.2; n = 13).

A total of 47.9% (n = 83) of the dentists said that the infrastructure at their workplace is incompatible with TD. 71.9% (n = 126) thought user-friendly software updates were needed, and 64.4% (n = 111) thought modern computers were required for future development. The highest need for using TD can be seen in Table 4.

**Table 4:** Missing requirements from dentists to use TD

*Missing requirements to use teledentistry*	*Number of participants*	*(%)*
***Allocated time***	111	64.4%
***Financial support***	111	64.3%
***Available technology***	95	55.2%
***Available professional material***	78	45.5%

The **digital dental index** is 14.24 (standard deviation (SD) = 5.5), and 81.2% received at least 10. In the analysis of socio-demographic variables and their impacts on the digital dental index, the following can be seen: gender has no significant relationship, and age has a significant effect, but the relationship is not linear. The highest value of the digital dental index can be seen among the 30-34-year-old group (mean = 16.6; M = 15.9), and the lowest value can be seen among the 55–59 age group (mean = 11.6; M = 11.9). The type of workplace also has a significant effect on the digital dental index; universities (mean = 16.2; M = 18) and national institutions (mean = 16.7; M = 16) have the highest value of the digital dental index, and the private healthcare providers (mean = 15.2; M = 14) and private companies (mean = 15.1; M = 16) has high importance. The lowest value of the digital dental index is experienced at public dental offices (mean = 11.3; M = 11). The settlement type of workplace also shows a similar, significant relationship; the value of the digital dental index is continuously decreasing from capital (mean = 16.8; M = 17) to villages (mean = 10.8; M = 9).

A linear regression model was executed to explain the impacts of the digital dental index. In this model, the dependent variable is the digital dental index; the independent variables are the number of patient needs, the perceived advantages and disadvantages of digital health, and the perceived need to use digital healthcare solutions. The model’s power is high (R-squared 0.249), and the perceived benefits positively affect the index. Still, age has a negative impact (the older, the less digital). The number of things needed to use digital healthcare solutions variable has no significant influence. It is important to emphasize that the value of the index is mainly influenced by how much demand they perceive from patients regarding the use of digital devices (Beta = 0,293) (Table 5).

**Table 5:** Digital dental index. A linear regression model was used with an enter variable selection method

	Unstandardized Coefficients		Standardized Coefficients		
	B	Std. Error	Beta	t	Sig.
(Constant)	14,614	1,896		7,706	0,000
How many solutions have you experienced need from your patients?	1,059	0,292	0,293	3,624	0,000
How many advantages do you see in digital healthcare solutions?	0,257	0,112	0,187	2,301	0,023
How many disadvantages do you see in digital healthcare solutions?	-0,112	0,199	-0,040	-0,565	0,573
How many things would you need to use digital healthcare solutions?	-0,002	0,161	-0,001	-0,013	0,989
Respondent age	-0,105	0,027	-0,279	-3,960	0,000

## Discussion

Dentists recognize certain advantages of TD, such as the time-saving potential achieved through decreased in-person visits and enhanced patient care quality.

Palmer et al. asked Canadian orthodontists to gauge their receptiveness to integrating TD in orthodontics. Most respondents agreed on the potential for digital technology to substantially enhance patient care and facilitate consultations among various specialists. These quality improvements promise to reduce healthcare disparities, particularly in rural and urban healthcare settings, and enable individuals in remote and isolated areas to access specialist care. Additionally, the framework of telemedicine opens avenues for the application of innovative technologies [12]. Similarly, Estai et al. surveyed dentists in Australia, yielding findings consistent with the earlier research. While most dental professionals viewed TD as a valuable tool for enhancing patient satisfaction, some expressed concerns about the reliability of digital tools [13]. Overall, there is an optimistic outlook on the concept of TD, with many practitioners endorsing its incorporation into their practice.

The growing prevalence of dental apps and the expanding realm of mobile health (M-health) further enrich the potential of TD. It provides an opportunity to develop structured and personalized prevention education programs, ultimately bolstering patient motivation. Moreover, TD allows for continuous monitoring and support of patients’ progress between appointments, improving patient care [14].

In Hungary, the digitalization of the dental sector primarily emphasizes digital workflows rather than doctor-patient communication. Interestingly, 12.8% of the respondents reported not using teledental solutions or related terminology. In contrast, foreign publications predominantly highlight the potential of telecommunication and telediagnosis in this context [15–17].

Regarding DCS, respondents expressed a significant concern about the inadequacy of infrastructure. Additional comments from respondents further underscore this issue, even though many are willing to allocate at least 20% of their working time to enhance their knowledge of TD. Recent survey findings point to broader opportunities and highlight the importance of continuous learning, including graduate and postgraduate education, in TD tools [18]. Palmer et al.‘s research also indicates that 54% of the respondents perceive infrastructure development as the most significant challenge.

Younger generations have a more comprehensive knowledge of DCS. Nevertheless, our analysis did not reveal any discernible differences in usage patterns based on age or gender. In Brazil, dental professionals with over a decade of experience in the field tend to be more receptive to using TD tools [19]. This is most likely due to more experience in dentistry. According to Estai et al., most healthcare professionals believe that TD is a valuable tool for enhancing patient satisfaction despite expressing concerns about the reliability of digital devices [5]. They are optimistic about TD and endorsing its integration into their practice. The proliferation of dental applications is increasingly evident, with a growing number of individuals utilizing them. TD offers the opportunity to establish a structured and personalized prevention education program, thereby reinforcing patient motivation. This approach enables continuous monitoring and progress support between appointments, improving patient care [14].

A total of 78.8% of the dentists recommended at least one available DDS to their patients. They recommend dental applications more often than they use them, which suggests that they (a) recommend applications without knowing the actual content and (b) recommend applications without adequate professional content control. In their recent research, Fernández et al. suggest that mHealth applications potentially enhance the oral health status of patients [14]. Widening dentists’ knowledge about teledental applications has the potential to lead to improved oral health status. Additionally, it is crucial to establish a professional overview to monitor the recommended applications, ensuring their suitability for personalized prevention and enhancing patients’ oral health literacy.

Engaging in patient education blogs can be a valuable source of information. Patients often play a significant role in their recovery and overall well-being. For example, well-known continuous glucose monitoring and insulin pump systems were introduced and developed based on patient feedback and input [20]. These blogs can provide much-needed support and guidance to individuals facing similar health conditions, such as those taking oral medications or dealing with autoimmune diseases [21]. However, a notable drawback of blogs authored by non-medical professionals is the potential for misinterpretation of medical reports provided by patients. Respondents have identified this as a significant issue.

Digital workflow tools like 3D planning, 3D printing, and 3D surgical templates are well-recognized, yet dentists use them only occasionally. However, dentists clearly desire to incorporate these technologies into their daily practice within the next three years. This trend also extends to other countries, where dentists express openness to embracing new TD applications in the future [22].

There is a significant difference between the capital and villages regarding adopting digital workflow tools. During dental digitalization, community and over-specialized dentistry decouple. Hungarian public dental offices use fewer digital tools, which could influence the separation between public dental health services and private dental care.

The digital dental index shows dentists want to incorporate TD tools in their daily practice. Notably, the index reflects that age exerts a negative influence, suggesting that the younger generation is more inclined to adopt digital solutions in their future dental practice.

Radiology and prosthodontics emerge as the predominant choices among dental specialties. The digital workflow aligns with the preferences of prosthodontics, particularly in areas like digital impression-taking and the digitalization of laboratory work phases. Digitalization can extend its benefits to other healthcare domains, as exemplified by Lee et al.‘s introduction of digital workflows in nursing, encompassing tasks like labeling, barcode scanning, and medication registration [23].

In dental radiology, the introduction of digital radiographs is often associated with teleradiology. However, digital radiographs are just one aspect of teleradiology. Artificial intelligence (AI) also plays a significant role [24]. Surprisingly, the survey results reveal that more than half of the respondents need more awareness of AI’s role in the dental field.

Online conferencing and e-learning platforms proliferated during the pandemic. After the lockdown, it was apparent to hold a global online conference to reach people worldwide [25]. Consequently, the knowledge and acceptance of online conferencing have increased.

Legal regulation of online platforms can ensure the safety of patients’ information [26]. New international legal guidelines could help to build confidence in dentists to use TD applications in the future. Colleagues should also know that protecting medical information via the Internet is confidential.

Our study marks a significant endeavor in unraveling the complexities of TD within the Hungarian dental context. The originality of our research stems from its distinct focus on investigating the awareness and adoption of various TD tools among Hungarian dentists. By offering comprehensive insights into the prevailing perceptions and utilization patterns, our study fills a crucial void in the existing literature, contributing original perspectives to the discourse on TD within the Hungarian dental landscape.

### Limitations

The main limitation of this study is the relatively limited number of completed questionnaires. It might be due to heightened online burdens resulting from the COVID-19 pandemic. Additionally, a significant number of surveys were found to need to be validated or completed, thus making them unsuitable for inclusion in the data assessment.

The outcomes show dentists’ current usage, knowledge, and recommendations. Our study was not designed to be representative initially. Instead, we addressed this limitation by applying weighting techniques, which allowed us to approximate our findings to the expected population.

### Implications and recommendations for future research

#### Bridging knowledge gaps in teledentistry

Our study illuminates significant gaps in the understanding and application of TD among Hungarian dentists. An essential revelation from our findings pertains to the limited definitions associated with TD, mainly focusing on digital communication solutions within dentistry. This restricted perception hinders the comprehensive utilization of various TD tools, leading to a division among practitioners—between those who do not employ any tools and those using multiple types. This stark divide underscores the need for a broader awareness and acceptance of the diverse TD tools available.

#### Embracing the diversity of teledentistry tools

One striking insight gleaned from our study is the openness of more than 70% of respondents toward incorporating digital dental tools in their practice. However, it is evident that the prevailing perception among participants predominantly links TD solely to digital workflows. This limited understanding must be more accurate in the breadth and potential of other TD tools beyond conventional applications. Emphasizing the array of TD tools available holds promise for significantly enhancing dental practices, offering time-effectiveness and cost-effectiveness, and ultimately improving patient care.

#### Practical implications for dental practice

Highlighting the diversity of TD tools is an academic exercise with substantial practical implications. It can influence dentists to explore and adopt a more extensive range of TD tools, thereby enhancing the efficiency and efficacy of their practice. This shift could lead to considerable improvements in patient care, resource optimization, and overall practice management.

#### Recommendations for future research and practice

Moving forward, we advocate for a comprehensive, representative study to generalize findings across the spectrum of Hungarian dentists. This would contribute significantly to the broader understanding and adoption of diverse TD tools. Such insights would be pivotal in shaping tailored training programs focused on introducing and familiarizing practitioners with the multifaceted landscape of TD tools.

In summary, our study’s revelations underscore the critical need to expand the scope of knowledge and practice in TD beyond traditional digital workflows. By bridging these gaps, future endeavors can propel Hungarian dental procedures toward embracing the full spectrum of TD tools, offering multifaceted benefits for practitioners and patients. Our study shows that TD’s current knowledge needs specific definitions, such as digital communication solutions in dentistry. We found that using TD tools is divided into not using or using more than one type, which creates a massive gap between dentists. Also, more than 70% of the respondents would like to use digital dental tools in the future.

## Conclusion

Dental care is a manual profession like surgery, but some treatments do not require a personal meeting. The main advantage of TD is that it provides space to create a calm environment and can rationalize patient pathways. Patients often feel white coat syndrome, anxiety, and fear. In addition, it is advantageous that the medical and dental history can be known in advance so that the doctor can wait for the patient’s first personal visit to the office, prepared and well-informed. It can be used effectively to monitor changes in the oral mucosa, develop dental confidence and proper oral care habits in children and adults, and even for orthodontic and periodontal monitoring. TD will increase opportunities for the profession, so developing and expanding our knowledge and learning new techniques is essential. Our survey suggests that it is necessary and inevitable to integrate this knowledge into both graduate and postgraduate education, which may form the basis of primary health care in the next decade. Applications and educational videos are also becoming a part of our everyday life in health promotion. Medicine based on telemedicine requires completely new skills and new experiences from doctors. They must have the appropriate knowledge and be prepared to solve the challenges caused by digital systems since quality control, professional expertise, and knowledge of technology are all essential for proper operation.

## Data Availability

The datasets used and analyzed during the current study are available from the corresponding author upon reasonable request.
